# Latent membrane protein 1 (LMP1) expression in Hodgkin lymphoma and its correlation with clinical and histologic parameters

**DOI:** 10.1186/s12957-017-1147-y

**Published:** 2017-04-20

**Authors:** Atif Ali Hashmi, Zubaida Fida Hussain, Kashif Ali Hashmi, Muhammad Irfan Zafar, Muhammad Muzzammil Edhi, Naveen Faridi, Mehmood Khan

**Affiliations:** 1 Department of Pathology, Liaquat National Hospital and Medical College, Karachi, Pakistan; 2 Department of Cardiology, Chaudhry Pervaiz Elahi Institute of Cardiology, Multan, Pakistan; 3 Department of Biostatistics, Liaquat National Hospital and Medical College, Karachi, Pakistan; 4 Department of Surgery, Brown University, Providence, RI USA; 5 Department of Medicine, Dhaka University, Dhaka, Bangladesh

**Keywords:** Hodgkin lymphoma, Epstein bar virus (EBV), Latent membrane protein 1 (LMP1), Pakistan

## Abstract

**Background:**

Hodgkin lymphoma is one of the most prevalent lymphoproliferative disorders in Pakistan; however, no risk factors for this disease have yet to be established in our population. Epstein–Barr virus (EBV) is a well-known risk factor for Hodgkin lymphoma in endemic regions of the world; however, frequency of its association in our population has not been widely studied. Latent membrane protein 1 (LMP1) expression by immunohistochemistry (IHC) is a surrogate marker of EBV in Hodgkin lymphoma. Therefore, we aimed to evaluate the frequency of expression of LMP1 in cases of Hodgkin lymphoma at our institute and its correlation with other clinical and histologic parameters.

**Methods:**

The study included 66 cases of Hodgkin lymphoma diagnosed at Liaquat National Hospital over a duration of 2 years from January 2014 to December 2015. The slides and blocks of all cases were retrieved, and representative blocks were selected for LMP1 by IHC. LMP1 expression of >10% of cells was considered as positive expression and correlated with histologic subtypes and clinical parameters like age, gender, and site of involvement.

**Results:**

The mean age of patients was 35.11 (+20.22). LMP1 expression was found in 68.1% (45/66) of cases of Hodgkin lymphoma. Mean age of the patients with LMP1 expression was 32.04 (+21.02). LMP1 expression was found in 40% cases of lymphocyte-rich, 66.7% of lymphocyte-depleted, 73.9% of mixed cellularity, 66.7% of nodular sclerosis, and 73.7% of classic Hodgkin lymphoma, NOS. No significant correlation of LMP1 expression with any clinical or histological parameter could be established in our studied patient population.

**Conclusions:**

A high frequency of expression of LMP1 is seen in cases of Hodgkin lymphoma at our setup comparable to endemic regions of the world; therefore, preventive and treatment protocols should be designed accordingly.

## Background

Hodgkin lymphoma (HL) is a neoplasm of germinal center and post germinal center B cells. It has a unique cellular composition which forms the basis of various histologic subtypes [1]. The association of HL and Epstein–Barr virus (EBV) was first demonstrated on the basis of increased frequency of HL in patients with history of infectious mononucleosis [2, 3] .This concept was further substantiated by detection of EBV in neoplastic Hodgkin Reed–Sternberg (HRS) cells [4–6]. Finally, a population-based cohort study established the increased relative risk of EBV-positive HL in patients with infectious mononucleosis [7].

HL is one of the most prevalent lymphoproliferative disorders in Pakistan; however, no risk factors for this disease have yet to be established in our population [8]. Although EBV is a well-known risk factor for HL in endemic regions of the world, frequency of its association in our population has not been widely studied. There are various methods of detecting EBV in HL including immunohistochemistry (IHC), in situ hybridization (ISH), and polymerase chain reaction (PCR) [9]. EBV-infected tumor cells express a subset of EBV genes including latent membrane protein 1 (LMP1), LMP2a, EBV nuclear antigen 1 (EBNA1), EBV small nuclear RNA transcripts (EBER), and the BamHI A region transcripts [10–12]. EBER and LMP1 are the two EBV markers most commonly used in epidemiologic studies in HL [13]. Therefore, we aimed to evaluate the frequency of expression of latent membrane protein 1 (LMP1) in cases of Hodgkin lymphoma at our institute and its correlation with other clinical and histologic parameters.

## Methods

**Fig. 1:**
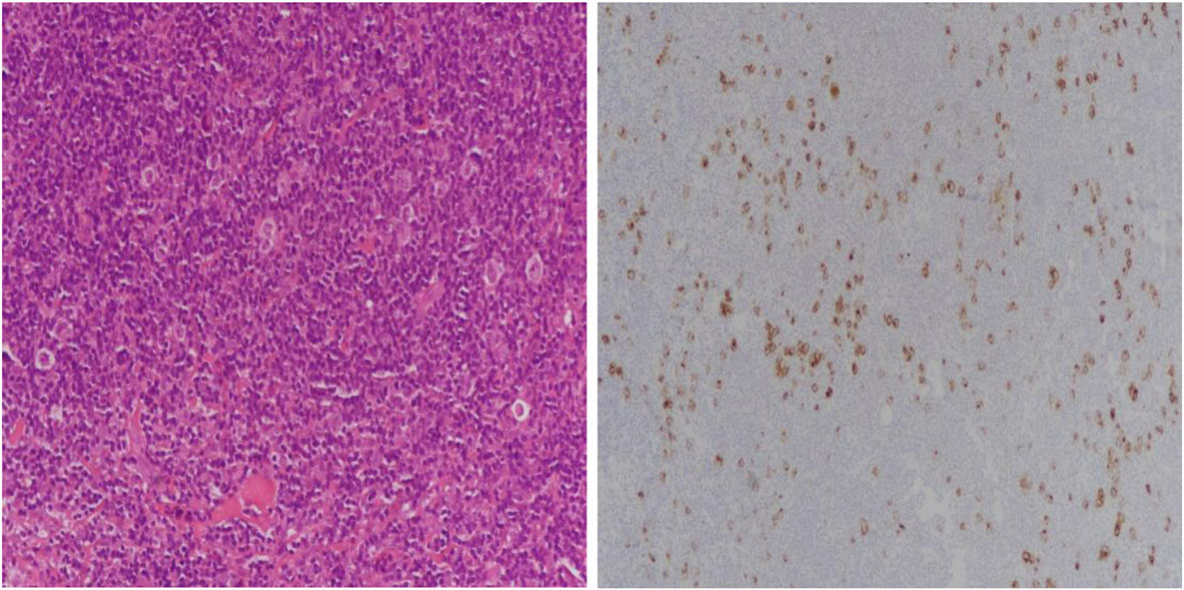
Mixed cellularity Hodgkin lymphoma with positive LMP1 expression noted in Reed–Sternberg cells

The study included 66 cases of Hodgkin lymphoma diagnosed at Liaquat National Hospital over a duration of 2 years from January 2014 to December 2015. The study was approved by the Institutional Ethical Review Committee of Liaquat National Hospital. The slides and blocks of all cases were retrieved and reviewed by two senior histopathologists with more than 5 years experience of reporting surgical pathology. One representative block of each case was selected for immunohistochemistry by LMP1. LMP1 expression of >10% of cells was considered as positive expression (Fig. 1) and correlated with histologic subtypes and clinical parameters like age, gender, and site of involvement.

## Results

**Fig. 2:**
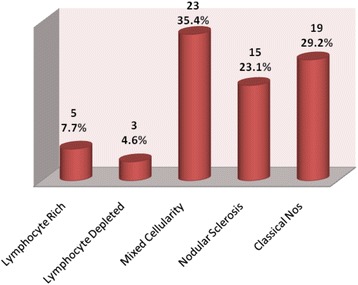
Distribution of subtypes of classic Hodgkin lymphoma

**Fig. 3:**
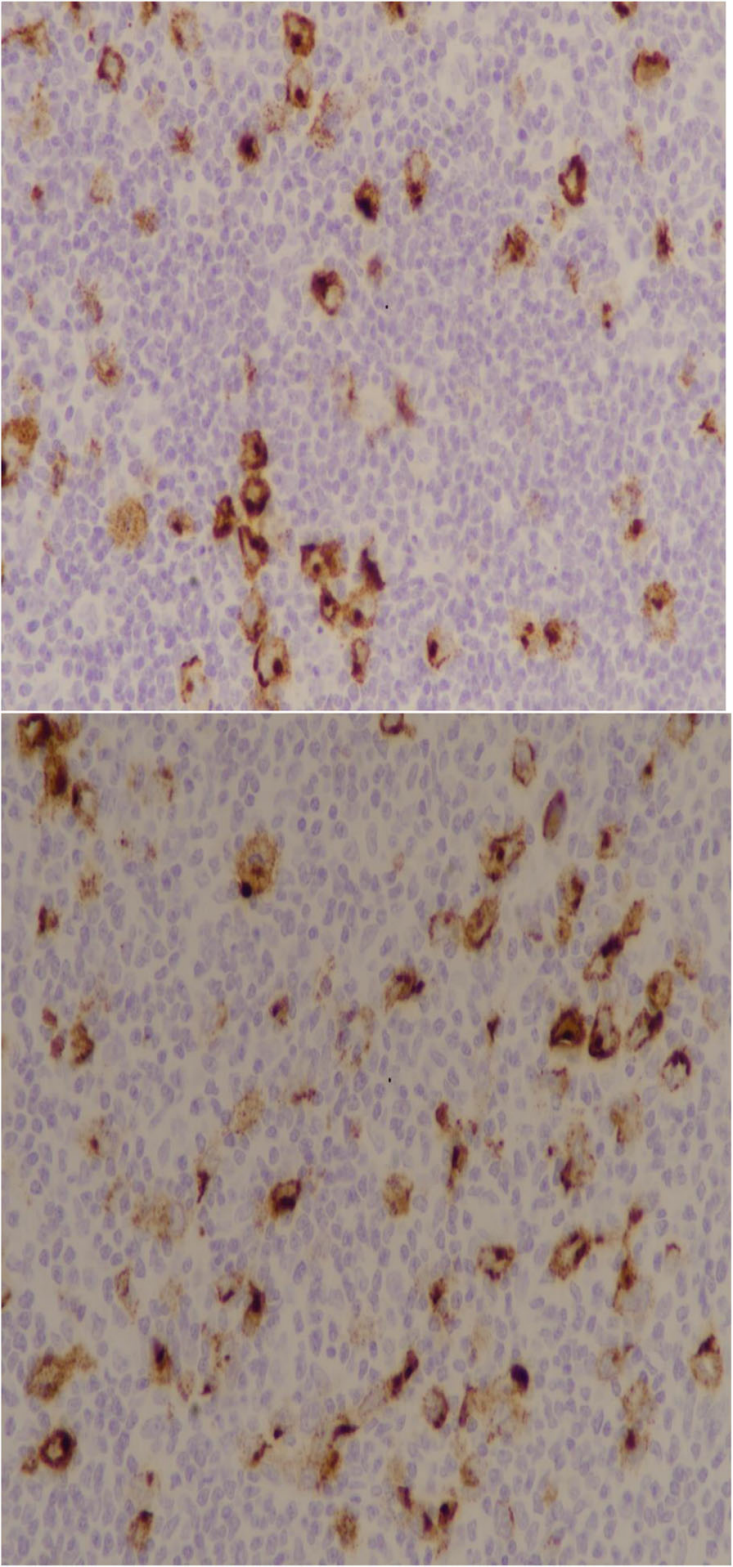
LMP1 expression in Hodgkin lymphoma. Reed–Sternberg cells are highlighted with LMP1 stain Golgi accentuation (×400 magnification)

The mean age of patients was 35.11 (+20.22). Male to female ratio was 2:1. 86.3% (57/66) cases were nodal, and mixed cellularity HL was the most common subtype accounting for 34.8% of cases (23/66) as shown in Fig. 2. LMP1 expression was found in 68.1% (45/66) of cases of Hodgkin lymphoma. Mean age of the patients with LMP1 expression was 32.04 (+21.02) while those with negative LMP1 was 41.67 (+17.03). No significant correlation was found between LMP1 expression and various demographic parameters (Table 1). LMP1 expression was found in 40% cases of lymphocyte-rich, 66.7% of lymphocyte-depleted, 73.9% of mixed cellularity, 66.7% of nodular sclerosis, and 73.7% of classic Hodgkin lymphoma, NOS (Fig. 3, Table 2).

**Table 1:** Demographic and clinical characteristics of patients involved in the study (*n* = 66)

	LMP positive *N* (%)	LMP negative *N* (%)	Total	*P* value
Age group				
≤30	23 (76.7)	7 (23.3)	30	0.435**
31–60	12 (61.3)	216 (38.7)	31	
>60	3 (60)	2 (40)	5	
Gender				
Male	32 (71.1)	13 (28.9)	45	0.455**
Female	13 (61.9)	8 (38.1)	21	
Site				
Nodal	37 (64.9)	20 (35.1)	57	0.252**
Extra nodal	8 (88.9)	1 (11.1)	9	
Diagnosis				
Classical Hodgkin	45 (69.2)	20 (30.8)	65	0.318**
NLPHL	0 (0)	1 (4.8)	1	

Chi square applied

**not significant at 0.05 levels

**Table 2:** LMP expression in subtypes of classic Hodgkin (*n* = 65)

	LMP positive *N* (%)	LMP negative *N* (%)	Total	*P* value
Lymphocyte rich	2 (40)	3 (60)	5	0.651**
Lymphocyte depleted	2 (66.7)	1 (33.3)	3	
Mixed cellularity	17 (73.9)	6 (26.1)	23	
Nodular sclerosis	10 (66.7)	5 (33.3)	15	
Classical NOS	14 (73.7)	5 (26.3)	19	

Chi-square applied

**not significant at 0.05 levels

## Discussion

In the current study, we evaluated the association of LMP1 expression in HL which is one of the most common lymphomas in Pakistan. Liaquat National Hospital is one of the largest tertiary care hospitals in Pakistan and represents patient population from both the urban and rural areas of the province. Various studies have demonstrated that EBV positivity in HL is associated with socioeconomic status [14]. Similarly, incidence of EBV-positive HL is higher among underdeveloped countries like Peru and Kenya as compared to developed countries like the USA and Europe [15–18]. This higher incidence of EBV-positive HL may be linked with underlying immunosuppression in underdeveloped regions or may be associated with timing of EBV infection. This later explanation is supported by a higher incidence of EBV-positive HL in children compared with HL in young adults. On the other, a second peak of EBV-positive HL is seen in older adults [19]. High incidence of EBV-positive HL in the elderly population is proposed to be associated with immunosuppression. We found a more homogenous expression of EBV in different age groups. Similarly, we did not find any significant association of EBV positivity with gender, although a few authors demonstrated a higher rate of EBV-positive HL in females as compared to male patients.

Table 3 compares the EBV protein expression in our study with other countries of South Asia [20–22]. Most studies show higher frequency of EBV expression as compared to our study. The highest incidence of EBV positivity was noted in India and Saudi Arabia (82 and 88%, respectively) [23, 24]. The lowest expression of EBV was noted in Jordan (43.6%) [25]. Our results were comparable with those of another study conducted in Pakistan depicting 50% expression of EBV in Hodgkin lymphoma [26].

**Table 3:** Comparison of EBV expression in Hodgkin lymphoma with other countries in South Asia

Country	Reference	Total positive cases of Hodgkin lymphoma	Lymphocyte rich	Lymphocyte depleted	Mixed cellularity	Nodular sclerosis
Korea	Park et al. [20]	17/25 (68%)	–	–	11/13 (85%)	5/8 (62%)
Saudi Arabia	Armstrong et al. [23]	7/8 (88%)	–	–	5/5 (100%)	2/2 (100%)
Pakistan	Fatima S et al. [26]	50/100 (50%)	0/6(0%)	–	41/57(71%)	19/35(54.2%)
China	Zhou XG et al. [21]	17/28 (60.7%)	–	0/1 (0%)	10/11(90.9%)	6/14(%)
India	Karnic S et al. [24]	82/100(82%)	–	–	–	43/50(86%)
Jordan	Sughayer MA et al. [25]	41/94(43.6%)	0/2(0%)		21/26(80.8%)	20/66(30.3%)
Malaysia	Peh SC et al. [22]	41/67(61.2%)	–	1/1 (100%)	27/31(87.1%)	12/33(36.4%)
Pakistan (our study)	Hashmi AA et al.	31/65(47.7%)	2/5(40%)	2/3(66.7%)	17/23(73.9%)	10/15(66.7%)

Various histologic subtypes of HL are defined on the basis of background lymphoid population, presence of fibrosis, relative percentage of HRS, and immunophenotype. In economically advanced countries like the USA, nodular sclerosis HL is the most frequent subtype of HL [27]. On the other hand, mixed cellularity HL is more frequent in the underdeveloped world. Only a few studies have been conducted in Pakistan with regard to HL. A regional study demonstrated the highest frequency of mixed cellularity HL accounting for 63% of all cases of HL and absence of bimodal age distribution as seen in our study [28].

Recent studies show that LMP1 expression may be used as a prognostic marker in some cases of Hodgkin lymphoma and therefore targeted therapy may be helpful [29]. Similarly, recent data suggests that EBV DNA expression in peripheral blood may be used as a clinical predictor of disease burden and monitoring response to therapy during disease course [30, 31].

As discussed above, EBV-infected HRS cells express a subset of EBV genes: LMP1, LMP2a, EBNA1, BamHI, and EBER. Studies have found a potential role of LMP1 and EBNA1 in tumor pathogenesis. EBNA1 expression is required for the replication and maintenance of the viral genome in dividing tumor cells [32]. Further role for EBNA1 in the pathogenesis of HL has not been determined. However, LMP1 and LMP2a appear to play a key role in the development of HL. HRS cells harbor crippling mutations in their immunoglobulin (Ig) genes, and due to lack of functional Ig, they are prone to die by apoptosis. Expression of LMP1 or LMP2a helps HRS cells to escape apoptotic mechanisms [33]. Various authors correlated the expression of EBV in HL with histological subtypes. Mixed cellularity HL is more likely to be EBV positive compared to nodular sclerosis HL [34]. A study conducted in Pakistan demonstrated 71% expression of EBV in mixed cellularity HL compared to 54% expression in nodular sclerosis subtype [26]. We also found a higher frequency of expression of LMP1 in mixed cellularity HL; however, the results are not statistically significant.

## Conclusions

A high frequency of expression of LMP1 is seen in cases of Hodgkin lymphoma at our setup comparable to endemic regions of the world; therefore, preventive and treatment protocols should be designed accordingly.

## Abbreviations

HL: Hodgkin lymphoma
LMP1: Latent membrane protein 1
